# PD09 - Association of the TNFα -308 G>A polymorphism with clinical phenotypes of asthma in Moldovan children

**DOI:** 10.1186/2045-7022-4-S1-P9

**Published:** 2014-02-28

**Authors:** Olga Cirstea, Liubov Vasilos, Ala Cojocaru, Dorina Savoschin, Tatiana Ivaschenko, Mikhail Aseev, Dumitru Amoasii

**Affiliations:** 1 Institute for Maternal and Child Healthcare, Chisinau, Moldova Republic; 2 Laboratory of Prenatal Diagnosis of Congenital and Inherited Diseases, D.O. Ott Research Institute, Saint Petersbur, Russia; 3 N. Testemitanu State Medical and Pharmaceutical University, Chisinau, Moldova Republic

## Background

Patients with asthma show a marked phenotypic variability, suggesting etiological heterogeneity and strong environmental influences. Bronchial asthma is determined by a complex interaction between genetic and environmental factors, but these mechanisms are not yet elucidated.

**The aim** of the study was to evaluate peculiarities of the TNFα gene polymorphism (-308 G>A) incidence and its association with different clinical evolution of bronchial asthma in Moldovan children.

## Material and methods

TNFα gene polymorphism (-308 G>A) was studied in 180 Moldavian children in order to assess the prevalence of the allelic variants of the gene in 90 children with asthma and 90 controls. All the patients underwent complex clinical and functional examination (spirography with bronchodilatational test), and laboratory evaluation (molecular genetics, immunological and general tests).

## Results

Following genotypes of the TNFα gene were identified - TNFα -308 G/G, TNFα -308 G/A and TNFα -308 A/A. The study showed that the homozygous genotype TNFα -308 G/G has protective role, being significantly more frequently identified in children with solitary form of asthma compared with those with allergic triad (86.2% versus 56.4%, respectively; OR = 4.83, 95% CI: 1.41 to 16.54, p<0.05). However, functionally compromised genotype TNFα -308 G/A was found more frequently in children with asthma associated with other allergic symptoms (ie., 38.5% compared with 13.8% in cases with asthma alone, p<0.05) and was observed twofold more frequently in boys with mild asthma with no associated atopic symptoms, compared to those with moderately to severe evolution of the disease (40.9% versus 20.7%, respectively; p> 0.05). Noticeably, the homozygous genotype TNFα -308 A/A group was identified only in children with allergic triad in 5.1% of cases.

## Conclusion

Study results demonstrate the association of the functionally compromised of genotypes of the TNF-α-308 gene with different phenotypes of asthma in Moldavian children.

